# Analysis of the coding sequence and expression of the coiled-coil α-helical rod protein 1 gene in normal and neoplastic epithelial cervical cells

**DOI:** 10.3892/ijmm.2012.877

**Published:** 2012-01-03

**Authors:** JOANNA PACHOLSKA-BOGALSKA, MAGDALENA MYGA-NOWAK, KATARZYNA CIEPŁUCH, AGATA JÓZEFIAK, ANNA KWAŒNIEWSKA, ANNA GOźDZICKA-JÓZEFIAK

**Affiliations:** 1 Department of Animal Physiology and Development, Adam Mickiewicz University, 61-614 Poznan; 2 Department of Biotechnology and Microbiology, Jan Dlugosz University, 42-218 Czestochowa; 3 Department of Molecular Virology, Adam Mickiewicz University, 61-614 Poznan; 4 Genesis Center for Medical Genetics, 60-601 Poznan; 5 Department of Gynaecology and Obstetrics, Medical University of Lublin, 20-081 Lublin, Poland

**Keywords:** cervical carcinoma, human papilloma virus, coiled-coil α-helical rod protein 1

## Abstract

The role of the CCHCR1 (coiled-coil α-helical rod protein 1) protein in the cell is poorly understood. It is thought to be engaged in processes such as proliferation and differentiation of epithelial cells, tissue-specific gene transcription and steroidogenesis. It is supposed to participate in keratinocyte transformation. It has also been found that this protein interacts with the E2 protein of human papilloma virus type 16 (HPV16). The oncogenic HPV forms, such as HPV16, are known to be necessary but not sufficient agents in the development of cervical carcinoma. In the present study, the *CCHCR1* gene coding sequence and its expression was analyzed in normal, precancerous and cervical cancer cells. Changes in the non-coding region were found in 20.3% of the examined probes from women with cervical cancer or precancerous lesions and in 16.67% of the control probes. Most of the detected changes were single nucleotide polymorphisms (SNPs). Changes in the coding region were found in 22.8% of the probes with cervical cancer and in 16.67% of the control probes and all of them were SNPs. The level of *CCHCR1* transcripts was determined using the real-time PCR method and the highest gene expression was detected in the H-SIL group and slightly decreased in the cervical carcinoma cells, compared with the control probes. It suggests that CCHCR1 could have a role in the process of cervical epithelial cell transformation, but this suggestion must be confirmed experimentally.

## Introduction

The oncogenic human papilloma virus (HPV) forms (HPV16, 18, 31 and 33) are etiological agents in the development of cervical carcinoma. However, the cell factors engaged in the process of the cancer development in cervical epithelial cells are poorly known (1,2).

Using the yeast two-hybrid system and the cDNA library from normal epithelial tissue, we have previously shown that the protein CCHCR1 (coiled-coil α-helical rod protein 1) interacts with the E2 protein of human papillomavirus type 16, which suggests its important role in the epithelial tissue function (3).

CCHCR1 is a protein of unknown role in the cell. This protein shows little homology with known proteins (4). Secondary structure predictions for the CCHCR1 protein suggests that it contains several segments of coiled-coil structure. CCHCR1 is either a nuclear or cytoplasmic protein, but was also found in mitochondria and endosomes (5), and also in keratinocyte pseudopodia *in vitro*. Its nuclear localization and possibility for the dimerization and interactions with DNA (via a leucine zipper motif) suggests a role of CCHCR1 in regulating cell differentiation or proliferation (6–8). CCHCR1 was found to be overexpressed in keratinocytes at psoriatic skin lesions, whereas in paired samples from normal appearing skin it was barely detectable. Therefore it was suggested that it could be involved in psoriasis susceptibility (4), but the connection of changes in the *CCHCR1* gene with psoriasis is still the subject of investigations (6,9). Functional analysis in the transgenic mouse model revealed that the CCHCR1 psoriatic allele is not enough to cause the psoriatic disease and additional genes or environmental stimulation is necessary to trigger off the psoriatic phenotype in the mouse (10,11).

The *CCHCR1* gene, encoding a 782 amino acid protein, is located on chromosome 6 and consists of 18 exons (5,12). The *CCHCR1* gene is highly polymorphic. It has 2 transcription start sites, which are located 79 and 430 bp upstream of the translation start site (ATG) in exon 2. Alternative transcription of the 5′-untranslated region can be regulated in a tissue by alternative usage of promoters (5).

The aim of the present study was the analysis of the *CCHCR1* gene in precancerous and cancer lesions and in HPV positive and negative dysplasia cells.

## Materials and methods

### Material

The investigations were performed using cervical cancer specimens obtained from women who were subjected to surgery during 2004–2008, because of histologically confirmed neoplastic lesions. The study material consisted of 73 patients that underwent surgeries due to: squamous cell carcinoma of the cervix or adenocarcinoma of the cervix and low- and high-grade squamous intraepithelial lesions. In respect to the differentiation of the neoplastic cells, the following groups of patients were identified according to the WHO classification system: G1 (n=10), G2 (n=16) and G3 (n=13) (Table I). According to FIGO clinical staging, 14 patients were classified as stage 0 (carcinoma *in situ*), 14 as IA, 22 as IB and 3 as IIA. The patients were between 39 and 61 years of age (mean 54.3). All patients underwent surgical procedures at the Department of Gynaecology and Obstetrics of the Medical University of Lublin. The control group was comprised of normal tissue of the uterine cervix obtained from 18 patients, 40–72 years of age, who underwent surgical treatment for uterine myomas.

Approvals obtained from the local Ethics Committee of Medical Universities in Lublin enabled us to collect cervical cancer specimens.

### DNA isolation

Genomic DNA was isolated from tissue samples using the QIAamp DNA Mini kit (Qiagen) according to the manufacturer’s indications.

### HPV analysis

Genomic DNA was used for amplification by PCR with two specific primer pairs complementary to the HPV genome, universal for 33 types of HPV viruses: MY09, 5′-CGTCCMARRGGAWACTGATC-3′ and MY11, 5′-GCMCAGGGWCATAAYAATGG-3′; for HPV16-E7/16A, 5′-ATAATATAAGGGGTCGGTGG-3′ and E7/16B 5′-CATTTTCGTTCTCGTCATCTG-3′; for HPV18 -ME18A, 5′-CACGGCGACCCTACAAGCTACCTG-3′ and ME18B, 5′-TGCAGCACGAATTGGCACTGGCCTC-3′. PCR reactions were performed in a total volume of 20 μl. The final mixture contained 1 μM primers, 200 μM dNTPs, 1X PCR buffer (10 mM Tris-HCl, pH 8.8 at 25°C, 1.5 mM MgCl_2_, 50 mM KCl, 0.1% Triton X-100) and 1 U/25 μl mixture of Taq polymerase (Fermentas). The samples were amplified for 35 cycles. Each cycle consisted of the following steps: denaturation at 95°C for 1 min (first cycle for 90 sec), annealing at 50°C for primer pairs MY09/11, 59°C for primer pairs E7/16A-E7/16B and 55°C for primer pairs ME18A/B, and extension at 72°C for 1 min. The reaction was performed in a DNA thermal cycler (Biometra). The amplification products were then analyzed on a 2% agarose gel with the addition of ethidium bromide in a UV transilluminator.

### CCHCR1 gene polymorphism analysis by PCR-SSCP and sequencing methods

Genomic DNA was used for amplification by PCR with two specific primers pairs complementary to fragments of the *CCHCR1* gene (sequence and localization of primers are provided in Table II). The PCR reactions were performed in a total volume of 20 μl. The final mixture contained 1 μM primers, 200 μM dNTPs, 1X PCR buffer (750 mM Tris-HCl, pH 8.8 at 25°C, 200 mM (NH_4_)SO_4_, 0.1% Tween-20), 1.5 mM MgCl_2_ and 1 unit/25 μl mixture of Taq polymerase (Fermentas). The samples were amplified for 35 cycles. Each cycle consisted of the following steps: denaturation at 95°C for 1 min (first cycle for 90 sec), annealing at proper temperature (Table III) for 30 sec and extension at 72°C for 1 min. The reaction was performed in a DNA thermal cycler (Biometra). Products of amplification were analyzed on a 2% agarose gel with addition of ethidium bromide in a UV transluminator. PCR products longer than 250 nucleotides were digested with endonucleases (Table II) before SSCP. Products of each PCR reaction (10 ml reaction mixture) were denatured chemically with formamide and thermically at 95°C for 15 min. Subsequently, denaturation samples were placed on ice. They were electrophoresed on a 10% polyacrylamide gel with 0.5X TBE buffer in 200 V for 12 h, stained with silver salts, and dried. PCR products were purified with a PCR purification mini kit (Qiagen) and then sequenced.

### CCHCR1 gene expression analysis

Cervical carcinoma, precancerous (L-SIL and H-SIL) and control tissues, collected during planned gynecological operations, were immediately put in RNAlater™ RNA stabilization reagent (Qiagen).

Total-RNA was isolated from cervical precancerous and cancer tissues and control cells using the RNeasy Mini kit (Qiagen) according to the manufacturer’s indications. RNA samples were treated with DNase I (Promega) and 1 μg of RNA (of each sample) was reverse-transcribed with SuperScript™ II RNaseH^−^ reverse transcriptase (Invitrogen) into cDNA using oligo(dT) primers. Real-time PCR was conducted in a Light Cycler Real-Time detection system (Roche Diagnostics) using SYBR^®^-Green I as the detection dye. Target cDNA was quantified using the relative quantification method. The quantity of the *CCHCR1* transcripts in each sample was standaridized by either glyceraldehyde-3-phosphate dehydrogenase (GAPDH) or RNA polymerase II (POL II) transcript levels. The RT-PCR reactions were performed in total volume of 20 μl. cDNA of 2 μl was added to an 18 μl mixture of LC-FastStart DNA Master SYBR-Green I, 1.5 mM MgCl_2_ and primers (sequence and localization of primers are given in Table III).

### Statistical analysis

The results obtained were analyzed statistically. Values of the analyzed parameters, due to the quotient scale of measurement, were characterized by average value, standard deviation and median, with the lower and higher quartile providing the changeability range. Due to the diagonal distribution of the studied parameters evaluated using the Shapiro-Wilk W test for the analyses of existence of differences, non-parametric tests were used. To discover differences between the compared groups, the Kruskal-Wallis H test was used to compare more than two groups and the Mann-Whitney U test to compare two independent groups. The 5% error in concluding was assumed, and P<0.05 indicated statistically significant differences.

### Methylation level analysis

Analysis of the DNA methylation level was made using the EZ DNA Methylation kit™ (Zymo Research) according to the manufacturer’s indications. Genomic DNA (0.5–1 μg) was used for the reaction of deamination. Deaminated DNA was used for the PCR reaction, with starters complementary to deaminated DNA: CCHCR1-BF 5′-TTTAAGTAGTGTTAGTTTGTG-3′ and CCHCR1-BR 5′-TCTTCATCTATCCCTTCACC-3′. The PCR reactions were performed in a total volume of 10 μl. The final mixture contained 1 μM primers, 200 μM dNTPs, 1X PCR buffer, 2 mM MgCl_2_ and 1 unit FastStart TaqDNA Polymerase (Roche Diagnostics) and 2 μl of deaminated DNA. The samples were amplified for 40 cycles. Each cycle consisted of the following steps: preliminary denaturation at 95°C for 5 min, denaturation at 95°C for 35 sec, annealing at 56°C for 45 sec and extension at 72°C for 1 min. Reaction was performed in a DNA thermal cycler (Biometra). Products of amplification were analyzed on a 2% agarose gel with addition of ethidium bromide in UV transluminator. The products 675 bp long were cut out of the agarose gel, eluted with MinElute Gel Extraction kit (Qiagen), according to manufacturer’s instructions and cloned into pGEM^®^ T-Easy Vector (Promega). DH5α *E. coli* were transformed with recombinant pGEM^®^ T-Easy. Clones with recombinant plasmid were selected, plasmids were isolated with QIAprep Spin Miniprep kit (Qiagen), according to the manufacturer’s instructions and then sequenced.

### Computer analysis

Programs MPromDb (http://bioinformatics.med.ohio-state.edu/MPromDb/index.jsp) and Cpgplot (http://www.ebi.ac.uk/emboss/Cpgplot) were used for computer analysis of the region located upstream the first transcription start site of the *CCHCR1* gene.

## Results

In DNA probes isolated from cervical cells, HPV was detected in 32 of 36 patients with squamous cell carcinoma, 11 of 14 with H-SIL, in all of those with adenocarcinoma and none of the control group women (Table IV). In the same DNA probes the *CCHCR1* gene was analyzed with PCR, SSCP and sequencing methods. The changes detected in the study region are present in Table V. Changes in the sequence of the *CCHCR1* gene were detected in the non-coding and coding region as well. Changes in the non-coding region were found in 20.3% of the examined probes from women with cervical cancer or precancerous lesions and in 16.67% of the control probes. Most of the detected changes were SNPs. Changes in the coding region were found in 22.8% of the probes with cervical cancer and in 16.67% of the control probes. These changes were detected in exons 3, 9, 12 and 15. All of identified changes were SNPs. Six types of changes (at sites 3169, 3171, 3189 and 3356 in exon 3; 9436 and 9437 in exon 9) were connected with the amino acid change and two others changes (at sites 12622 in exon 12 and 14494 in exon 15) were not.

**Figure 1:**
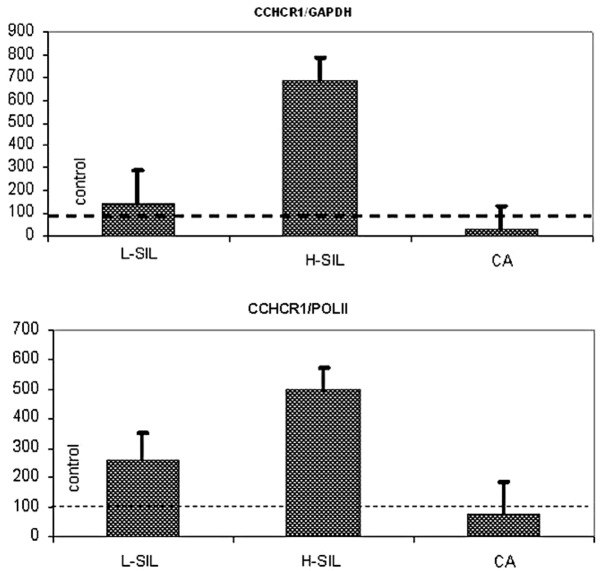
The expression of CCHCR1 in L-SIL, H-SIL and cervical cancer (CA) tissues. The quantity of the *CCHCR1* transcripts was standardized by glyceraldehyde-3-phosphate dehydrogenase (GAPDH) and RNA polymerase II (POL II) transcript levels.

The mRNA *CCHCR1* levels were determined by a real-time PCR method with RNA probes isolated from cervical precancerous, cancer and control cells. RT-PCR results are presented as a percentage of their controls at Fig. 1 and in Table VI. The highest *CCHCR1* transcripts values were detected in the H-SIL group. Interestingly, *CCHCR1* gene expression was slightly decreased in cervical carcinoma cells, compared with control probes.

According to the Kruskal-Wallis H test, statistically significant differences were found in *CCHCR1* transcripts levels between the compared groups (H=9.06; P=0.03 for CCHCR1/GAPDH and H=9.23; P=0.03 for CCHCR1/POL II). Intergroup analysis revealed differences between the control group and H-SIL and H-SIL and CA (P=0.005 for CCHCR1/GAPDH and P=0.01 for CCHCR1/POL II).

**Figure 2:**
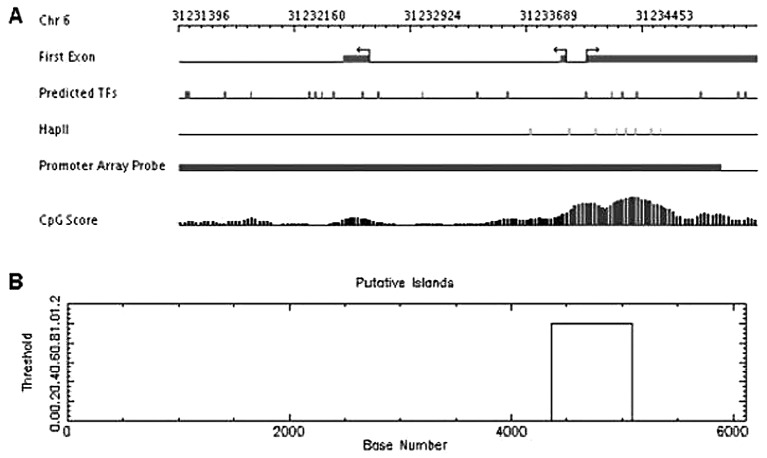
Computer analysis of the region located upstream of the first transcription start site of the *CCHCR1* gene performed using the programs: (A) MPromDb (http://bioinformatics.med.ohio-state.edu/MPromDb/index.jsp) and (B) Cpgplot (http://www.ebi.ac.uk/emboss/Cpgplot).

Because there is no information in the literature about the structure and function of the regulatory region of the *CCHCR1* gene we decided to perform computer analysis of the region located upstream of the first transcription start site using the programs MPromDb and Cpgplot. We analyzed a DNA fragment about 6 kbp long and found a CpG island, about 1 kbp long, located just above first transcription start site of the *CCHCR1* gene (Fig. 2).

Therefore, we performed the preliminary analysis of methylation level of the 647 bp DNA fragment with 55 CG pairs within. For this analysis we used DNA isolated from cervical epithelial cells collected from 3 healthy women (control probes) and from 3 women with carcinoma planoepitheliale. We did not notice any differences in the DNA methylation profile in the control and cervical probes. In the analyzed DNA fragment all CG pairs were not methylated.

## Discussion

The function of the CCHCR1 protein, is poorly known. It is most probably engaged in the control of epithelial cell proliferation (4,8,13). It is also involved in processes like tissue-specific gene transcription (14), steroidogenesis (5) and probably metabolism of steroids (13). Suomela *et al* (15) have suggested that CCHCR1 plays a role in keratinocyte transformation. Our previous analysis revealed that CCHCR1 interacts with the protein E2 of the human papillomavirus type16 (HPV16) in normal cervical epithelial cells (3). E2 is the main viral regulatory protein. It plays a significant role, among other things, in regulating the expression of other viral proteins (16,17).

The *CCHCR1* gene is highly polymorphic (4,6,10,12). In the genome of psoriatic persons some mutations were identified, which could probably change the secondary structure of this protein and influence its biochemical properties and antigenicity (7,8). We also demonstrated that the *CCHCR1* gene in the non tumor and tumor cells of the cervical epithelium is highly polymorphic. Almost all detected changes, in the non-coding as well as in the coding region, were single nucleotide polymorphisms (SNPs) characterized in databases (www.ncbi.nlm.nih.gov/SNP/). Obtained results did not allow us to identify changes which were characteristic of the cervical cancer patients. Our analyses were performed in a relatively small research group and did not allow for the reliable evaluation the *CCHCR1* genotype frequency. It would be intentional to expand the research group to confirm or exclude if a particular genotype has a higher frequency in a population of ill women compared to a control group.

Tiala *et al* (13) suggest, that for the pathogenesis of such diseases like psoriasis the amount of protein produced in the cell could also be important. Very small differences in the experssion of the *CCHCR1* gene in basal kerationcytes could influence the local production of steroids (13,18). SNPs in the *CCHCR1* gene could have an impact on the amount of produced protein, through an influence on the mRNA stability, gene expression or different regulation of this gene in response to environmental stress (13). In this context nucleotide changes in the non-coding region may have an influence on the regulation of *CCHCR1* gene expression, but this suggestion need to be experimentally confirmed.

*CCHCR1* gene expression analysis revealed that *CCHCR1* mRNA levels were higher in L-SIL and H-SIL probes compared to those in normal cervical cells. The highest *CCHCR1* mRNA levels were observed in H-SIL probes, whereas the levels were lower in cervical carcinoma compared to control probes. The differences were statistically significant.

Overexpression of the *CCHCR1* gene could stimulate the proliferation of cervical carcinoma cells at the early stages of neoplasia. Suomela *et al* (15) found that proliferative cells of cutaneous squamous cell cancer (SCC) at the invasive front expressed CCHCR1, whereas the cohesive cancer cells in the middle were CCHCR1-negative. CCHCR1 expression was associated with positive EGFR staining. Cells positive for the hyperproliferation marker Ki67 were located mostly in the same areas as CCHCR1-positive cells. However, in grade III SCCs Ki67 was more abundantly present in cohesive tumor areas that were devoid of CCHCR1 expression. CCHCR1 expression was not induced *in vitro* in the most aggressive and metastatic SCC cell lines. It was also found that with ascending tumorigenicity and proliferative state, *CCHCR1* mRNA was downregulated in HaCaT cells. In contrast to benign KC hyperproliferation in psoriasis, the hyperproliferation marker Ki67 expresion *in vivo* and *in vitro* was associated with CCHCR1 expression in malignant transformation. CCHCR1 may participate in regulating cell proliferation only to a certain point in oncogenesis (15).

Little is known about the regulation of the *CCHCR1* gene expression and the structure of its regulatory region. For that reason we performed computer analysis of the region located upstream of the first transcription start site which is probably of great importance as far as the regulation of *CCHCR1* expression is concerned. The analysis revealed that the CpG island, about 1 kbp long, is located just above the first transcription start site of the *CCHCR1* gene.

CpG islands are regions of higher frequency of CpG compared to the rest of the genome. CpG islands are protected from DNA methylation in normal cells, at all stages of development and in all types of tissues by mechanisms, which are poorly understood (19,20). These regions function as strong promoters and can initiate DNA replication as well (19).

A lot of changes occurring in cancerous cells are caused by genetic and epigenetic abnormalities. The genome can undergo simultaneous general hypomethylation and regional hypermethylation of CpG islands, which can result in the occurrence of conditions leading to tumor development. This phenomenon can result in silencing of tumor suppression genes, loss of gene imprinting, and less probably, oncogene activation through demethylation, increase of the frequency of point mutations in methylated CpGs and instability of microsatellite DNA (20–23). However, there is no consensus as to the direct role of DNA methylation in carcinogenesis.

We analyzed the region located upstream of the first transcription start site of the *CCHCR1* gene. We observed no differences in the methylation level between carcinoma planoepitheliale and the normal probes. In the analyzed region all CG pairs were not methylated. Even though the analysis was carried out in few probes, we can assume that mechanisms other than methylation of the region with the probable regulatory function are responsible for the changes in *CCHCR1* mRNA levels.

Continuation of research on this subject seems to be very interesting and important, particularly in relation to the CCHCR1 and the HPV16 E2 protein interactions. Recent reports suggest that CCHCR1 can act as the negative regulator of keratinocyte proliferation and that inappropriate function of CCHCR1 protein can lead to incorrect keratinocyte proliferation (11) and transformation (15).

## Figures and Tables

**Table I:** The WHO and FIGO classification of patients with tumors of the uterine cervix.

	N
FIGO classification	
0	14
IA	14
IB	22
IIA	3
Total	53
WHO classification	
G1	10
G2	16
G3	13
Total	39
Squamous cell carcinoma	
Keratizing	22
Non-keratizing	10
Basaloid	4
Total	36
Adenocarcinoma	
Endocervical	1
Endometroid	1
Clear cell	1
Total	3

**Table II:** Primers used to PCR-SSCP study of the *CCHCR1* gene.

Exon	Region of amplification (according to AB088104)	Fragment length (nt)	Primer sequence 5′→3′	Annealing temperature (°C)	Restriction enzyme
1a	91–512	422	CCACTATGTGTTAGGACTCGAG GATTCTGGGCAGTGCCTTTACC	56.0	*Pvu*II
1b	765–1174	410	GTGTCTTTGTTTCTCCTCTTGTCC GAAAACGGCGTGGATGGATCCC	57.0	*Alu*I
2	812–1170	359	CAGAATCTAGAGCCTTCAAATAATGTG ACGGCGTGGATGGATCCCTA	55.5	*Alu*I
3	3071–3422	372	ACCTGCACTAACCTGTCTTTGA AATCCTTTCTACCCCTGCATTC	53.0	*Pst*I
4/5	6760–7204	445	GAGCCCCCTCTTCTTTCCGC CACAGATACATTCCTGCACCCTC	57.5	*Pst*I
6	7317–7471	155	GGCTGCTTTCCTCTGCCCGC GGGTCTGGGGGTTGGGCTGT	60.0	-
7	7666–7862	197	TTCTCCCACTCCTTCTCCCTC CGGGAGAAAAGAGAGTGCAGTG	56.5	-
89	9153–9522	370	GCCCAGCTCTCTCTCCTCC CCCACCCCTCCATCCCTGAT	57.5	*Ava*II
10/11	12065–12473	409	ATCAGTGACTTGTGCCCTCTC CACCTCAAAGTGC(AC)CAAACTTC	54.0	*Ava*II
12	12569–12765	197	CTGACTCTTTCTCTTCCCCGT CTCATCCTCTCCACCCTCTG	55.5	-
13/14	12815–13240	426	TCCTTTTAGGGGAGGCAGAG GAAGGCCCTATCCACCCTG	54.5	*Taq*I
15	14462–14656	195	CTGTGCCTTGGCCTCTCTGT GTCTGCCCTCCTGTCTCCTA	55.5	-
16	14725–14964	240	GGCTCTATCCGGGCTAGG TCCCTTGTCCCTTTGTGCTTG	54.5	-
17	15328–15171	178	CTTTCCCTCCAACTGTCAGC CTGGTGCTCATCTGCTGTCTT	54.0	-

**Table III:** Primers used to real-time PCR study of the *CCHCR1* gene.

	Fragment length	Primer sequence (5′→3′)	Annealing temperature
CCHCR1, F	204 bp	TGCGTGCTGCTTTGGCTGG	60°C
CCHCR1, R		CCCCTGCTCTTCTGGTTTC	
GAPDH, F	106 bp	CAATGACCCCTTCATTGACC	60°C
GAPDH, R		GACAAGCTTCCCGTTCTCAG	
POL II, F	163 bp	GCAAATTCACCAAGAGAGAC	60°C
POL II, R		ATGTGACCAGGTATGATGAG	

**Table IV:** Study groups and frequency of human papilloma virus (HPV) type 16 and/or 18 DNA occurrence.

Group	No. of cases	HPV oncogenic types 16/18	% HPV positive
L-SIL	6	3	66.67
H-SIL	14	8	78.57
Squamous cell carcinoma	36	26	88.89
Adenocarcinoma	3	1	100
Control	18	0	0

**Table V:** Changes detected in the coding sequence of the *CCHCR1* gene.

	Change in protein sequence^a^					
			
Change in DNA sequence^b^	Change	Amino acid position	Position in codon		Number of probes^c^	Recognition
246 (c→t) exon 1a		Non-coding sequence		SNP	3	Carcinoma planoepitheliale
					2	CIN3
					2	Control
251 (g→c) exon 1a		Non-coding sequence		-	1	Carcinoma planoepitheliale
394 (g→t) exon 1a		Non-coding sequence		-	1	Carcinoma planoepitheliale
808 (a→g) exon 1b		Non-coding sequence		SNP	4	Carcinoma planoepitheliale
					1	CIN3
					1	Control
3169 (g→a) exon 3	CGG(Arg)→CAG(Gln)	102	2	SNP	1	Carcinoma planoepitheliale
					1	CIN3
					1	Control
3171 (c→t) exon 3	CGG(Arg)→TGG(Trp)	103	1	SNP	2	Carcinoma planoepitheliale
					1	CIN3
					3	Control
3189 (c→t) exon 3	AGG(Arg)→AGC(Ser)	109	3	SNP	2	Carcinoma planoepitheliale
					1	CIN3
					2	Control
3356 (g→c) exon 3	AGG(Arg)→AGC(Ser)	164	3	SNP	2	Carcinoma planoepitheliale
					3	CIN3
					3	Control
9436 (c→t) exon 9	CGT(Arg)→TGT(Trp)	416	1	SNP	2	Carcinoma planoepitheliale
					1	CIN3
9437 (g→a) exon 9	CGG(Arg)→CAG(Gln)	417	2	SNP	1	Carcinoma planoepitheliale
12622 (t→c) exon 12	GAT(Asp)→GAC(Asp)	500	3	SNP	1	Carcinoma planoepitheliale
					1	Adenocarcinoma
					1	CIN3
14494 (g→a) exon 15	TTG(Leu)→TTA(Leu)	637	3	SNP	1	Carcinoma planoepitheliale

a Change in protein sequence according to NCBI accession no. NP_061925;

b Change in DNA sequence according to NCBI accession no. AB088104;

c Number of probes in which change was detected.

**Table VI:** Expression of *CCHCR1* gene, real-time PCR data.

A. Comparison of the CCHCR1/GAPDH values in the control, L-SIL, H-SIL and CA groups					
Group	Mean	Median	25th percentile	75th percentile	Range
Control group	0.0061	0.00048	0.00033	0.00066	0.00032–0.034
L-SIL	0.009	0.0036	0.00034	0.0123	0.00034–0.034
H-SIL	0.042	0.036	0.0075	0.076	0.0065–0.090
CA	0.0021	0.0015	0.00047	0.0029	0.00034–0.0065

B. Comparison of the CCHCR1/POL II values in the control, L-SIL, H-SIL and CA groups					

Group	Mean	Median	25th percentile	75th percentile	Range

Control group	0.104	0.0162	0.012	0.047	0.0104–0.524
L-SIL	0.270	0.233	0.026	0.456	0.0245–0.649
H-SIL	0.522	0.509	0.221	0.823	0.101–0.968
CA	0.082	0.0506	0.022	0.110	0.00625–0.284

## Abbreviations

CCHCR1: coiled-coil α-helical rod protein 1
HPV: human papilloma virus
H-SIL: high-grade squamous intraepithelial lesions
L-SIL: low-grade squamous intraepithelial lesions
SNP: single nucleotide polymorphism
SSCP: single stranded conformation polymorphism
